# Pre-pregnancy BMI, gestational weight gain and birth outcomes in Lebanon and Qatar: Results of the MINA cohort

**DOI:** 10.1371/journal.pone.0219248

**Published:** 2019-07-02

**Authors:** Mariam Ali Abdulmalik, Jennifer J. Ayoub, Amira Mahmoud, MINA collaborators, Lara Nasreddine, Farah Naja

**Affiliations:** 1 Primary Health Care Corporation, Doha, Qatar; 2 Nutrition and Food Sciences Department, Faculty of Agriculture and Food Sciences, American University of Beirut, Beirut, Lebanon; 3 Public Health Department, Ministry of Public Health, Doha, Qatar; Texas A&M University College Station, UNITED STATES

## Abstract

Accumulating evidence has highlighted the role of maternal nutritional status on fetal development, birth outcomes and child health. The Mother and Infant Nutritional Assessment (MINA) cohort is a 3-year follow-up study of pregnant women and their children in Qatar and Lebanon. This study reports on the characteristics and determinants of pre-pregnancy BMI and Gestational Weight Gain (GWG) of MINA particiants, as well as birth outcomes. A total of 272 pregnant women were recruited during their first trimester from primary healthcare centers as well as private clinics in Beirut (n = 194) and Doha (n = 147). During the first visit, data collection included pre-pregnancy weight, sociodemographic and lifestyle characteristics. The weight before delivery and neonatal outcomes were extracted from the medical records. GWG was calculated as the difference between weight before delivery and pre-pregnancy weight and was classified into insufficient, adequate, and excessive, as per the IOM criteria. Overall, 42.1% of women had a pre-pregnancy BMI≥25 Kg/m^2^ (58% in Qatar vs 30.8% in Lebanon, p<0.001). Only 30.2% of women had adequate GWG, while 25.7% and 44.1% of women had insufficient and excessive GWG, respectively. In the cohort 68.7% of infants had a weight adequate-for-gestational age (AGA), 6.7% were SGA and 24.6% were LGA. The proportions of LGA were higher with greater GWG (p<0.05). After adjustment, Qatari women were 3 times more likely to be overweight or obese before pregnancy while a higher education level was associated with significantly lower odds of pre-pregnancy BMI≥25 Kg/m^2^. Pre-pregnancy BMI≥25 Kg/m^2^ and regular breakfast consumption were predictors of excessive GWG (OR: 3.20, CI: 1.48–6.91; OR: 2.84, CI: 1.15–7.02, respectively). The high prevalence of pre-pregnancy overweight and excessive GWG among MINA participants underscores the need for culture-specific intervention programs to promote healthy body weight in women of childbearing age, and prevent excessive weight gain during pregnancy.

## Introduction

Countries of the Eastern Mediterranean Region (EMR) are witnessing the nutrition transition with its characteristic shifts in diet, lifestyle, and alarming surges in obesity and nutrition-related chronic diseases [1]. Of particular concern amidst these ongoing changes is the nutritional status of women of childbearing age, given its potential impact not only on the health of mothers but also on the offspring’s growth, development and disease susceptibility later in life [2]. The hypothesis of the Developmental Origins of Health and Disease (DoHaD), which is often coined as ‘fetal or metabolic programming’, refers to the adaptive physiologic and metabolic processes that occur during critical prenatal, perinatal and early postnatal periods, in response to environmental stressors such as suboptimal maternal nutritional status [3]. In the EMR, high rates of overweight and obesity have been reported among women of childbearing age, with estimates reaching as high as 79% [4–6], implying that women begin their pregnancy with a higher body mass index (BMI) [7]. The high pre-pregnancy BMI was in fact reported as a risk factor for pregnancy and obstetric complications, such as gestational diabetes mellitus, preeclampsia, and cesarean deliveries [8], as well as adverse neonatal outcomes such as perinatal mortality, macrosomia, and congenital anomalies [9, 10]. Pre-pregnancy BMI was also reported as one of the factors that influence gestational weight gain (GWG) and its adequacy, which in turn may affect the child’s growth pattern and risk for disease later in life [11–13]. In 2009, the Institute of Medicine published the revised GWG guidelines, providing specific weight gain recommendations based on pre-pregnancy BMI categories [14]. GWG below the recommendations was reported to increase the risk for low birthweight (LBW), impaired fetal growth and preterm births [15–17], while excess GWG was found to be associated with gestational hypertension, gestational diabetes mellitus, preeclampsia, complicated deliveries, macrosomia [15, 18, 19] as well as adverse cardiometabolic profile in the offspring [20, 21]. Despite the significant health impact of pre-pregnancy BMI and GWG on the health of both the mother and the child, and their implications on the burden of non-communicable diseases (NCDs), little is known about their prevalence and determinants in countries of the EMR. This is of particular importance to the region, where approximately 60% of mortality is attributed to NCDs [22]. To move this agenda forward, a collaborative endeavor was initiated between Lebanon and Qatar to launch the first mother and child cohort in the region, examining the impact of maternal nutritional status and lifestyle characteristics on neonatal outcomes [23], and investigate the association of nutrition imbalances early in life with birth outcomes, growth patterns, as well as early determinants of NCDs [23]. The “Mother and Infant Nutritional Assessment” (MINA) cohort, a 3-year follow-up study of pregnant women and their children, was launched in 2015 in two Arab countries of the EMR, the first representing middle-income fossil fuel-importer countries (Lebanon) and the second representing high income fossil fuel-exporters (Qatar) [23]. This study is the first to report on findings stemming from the MINA cohort in Lebanon and Qatar. It aims at 1) describing the socioeconomic and lifestyle characteristics of the MINA cohort participants in Lebanon and Qatar; 2) characterizing and examining the determinants of pre-pregnancy BMI and GWG in the cohort of pregnant women in Lebanon and Qatar and 3) presenting the cohort’s birth outcomes. A secondary objective of this paper is the evaluation of the socio-demographic determinants of loss to follow-up in the MINA cohort.

## Methods

This is the first study to report on the results of the MINA cohort, conducted in Beirut, Lebanon and Doha, Qatar. The details of the MINA protocol are published elsewhere [23]. In brief, the MINA cohort is a three–year follow-up study of pregnant women and their children. Women were recruited during their first trimester (0–13 weeks of gestation), between November 2015 and December 2018. Recruitment sites were the primary healthcare centers as well as private clinics in both Beirut and Doha. To be included in the study, women ought to be Lebanese, Qatari, or non-Qatari living in Qatar for more than 5 years, pregnant with a singleton, and not suffering from any chronic diseases that may affect dietary intake. Data collection for the MINA cohort took place at 9 time points/visits (3 visits during each trimester of pregnancy and 6 visits post-partum). In addition to these visits, delivery data were obtained through extraction from the hospital medical records. For the purpose of this study, data obtained from visit 1 and from medical records were used. The protocol of the MINA was approved by the Institutional Review Board at the American University of Beirut (Protocol ID: NUT. FN. 12) and the Primary Health Care Corporation in Qatar (Protocol ID: PHCC/ RC/15/04/006). All subjects signed a written consent before participating.

### Data collection

For visit 1, which took place during the first trimester of pregnancy, trained research assistants approached potential participants in the healthcare facility’s waiting room and introduced the study objective and protocol. After consent and during a face-to-face interview, the participants completed a multi-component socio-demographic and lifestyle questionnaire. In addition, following the interview, the participants’ height was measured.

The socio-demographic section included questions regarding age (in years), number of children (0, ≥1), education level (up to high school-including technical diploma, university or higher), employment status (employed, housewife), family ties with husband (yes, no), family income (low (<1000 USD), middle (between 1000 and 2000 USD), high (≥2000 USD)), husband’s age (in years) and husband’s education level (up to high school, university or higher). Lifestyle characteristics were pre-pregnancy smoking (current smoker, non-smoker-including past smoker), smoking during pregnancy, folic acid supplementation pre-pregnancy (yes, no) and during pregnancy, breakfast consumption (regular, non-regular) before and during pregnancy, as well as physical activity during pregnancy. The latter was assessed using the Pregnancy Physical Activity Questionnaire (PPAQ) [24]. Total physical activity was calculated by weighting each type of activity by its energy requirements defined in MET-minutes (multiples of the resting metabolic rate for an activity multiplied by the minutes performed). Three categories of physical activity, including low, moderate, and high intensity were assigned to the 1^st^, 2^nd^ and 3^rd^ tertile of METS-min per week. In addition to socio-demographic and lifestyle information, participants were asked to report their pre-pregnancy weight (Kg). Height was measured to the nearest 0.1 cm using standard protocol using the Seca 213 Stadiometer. In this study, pre-pregnancy BMI was calculated as the pre-pregnancy weight (in Kg) divided by the square of height (in m^2^).

The delivery and birth outcome data were extracted from the participants’ medical record at the hospital where delivery took place (after subjects’ consent). The data included the following variables:

- Weight before delivery (Kg): When admitted for delivery at the hospital, the weight of pregnant women was recorded.
- Neonatal outcomes including gestational age (weeks), birthweight (Kg), and length at birth (cm). Preterm delivery was defined as birth at < 37 completed weeks of gestation. LBW was defined as birthweight < 2.5 Kg, whereas fetal macrosomia was defined as birthweight > 4 Kg. In addition, birthweights were converted to gestational age and sex-specific percentiles. Birthweights below the 10^th^ centile were classified as small-for-gestational age (SGA) [25, 26], and those above the 90^th^ centile as large-for-gestational age (LGA) [27, 28].

Using data from visit 1 and from the medical record, GWG was calculated (Kg) as the difference between the weight before delivery (from the medical record) and the pre-pregnancy weight reported by the participant at visit 1. The 2009 Institute of Medicine recommendations were used to classify GWG into insufficient, adequate, and excessive [14], as follows: Insufficient GWG was defined as a weight gain during pregnancy of < 12.5 Kg in underweight women, < 11.5 Kg in normal weight women, < 7 Kg in overweight women, and < 5 Kg in obese women; adequate GWG was defined as a weight gain during pregnancy of 12.5–18 Kg in underweight women, 11.5–16 Kg in normal weight women, 7–11.5 Kg in overweight women, and 5–9 Kg in obese women; while excessive GWG was defined as a weight gain during pregnancy of > 18 Kg in underweight women, > 16 Kg in normal weight women, > 11.5 Kg in overweight women, and > 9 Kg in obese women.

### Statistical analysis

Characteristics of the study population were presented using descriptive statistics including means, SD and proportions. Independent t-test and chi-square tests were used to compare continuous and categorical variables (respectively) between the Lebanese and Qatari arms of the cohort. Simple and multiple logistic regression analyses were conducted to calculate the OR and their corresponding 95% confidence intervals describing the associations among socio-demographic and lifestyle variables with two main dependent variables: pre-pregnancy BMI and GWG. For GWG, multinomial logistic regressions were used with the reference category being “within recommendations”. Binary logistic regressions were used for pre-pregnancy BMI. In the multiple regression analyses, adjustments were made for those variables, which were significantly associated with the outcome in the simple regressions. Pairwise deletion was used to deal with missing data. Statistical analyses were carried out using Statistical Package for Social Sciences (SPSS) software (SPSS Inc., Chicago, IL). P-values less than 0.05 were considered statistically significant.

## Results

**Fig 1:**
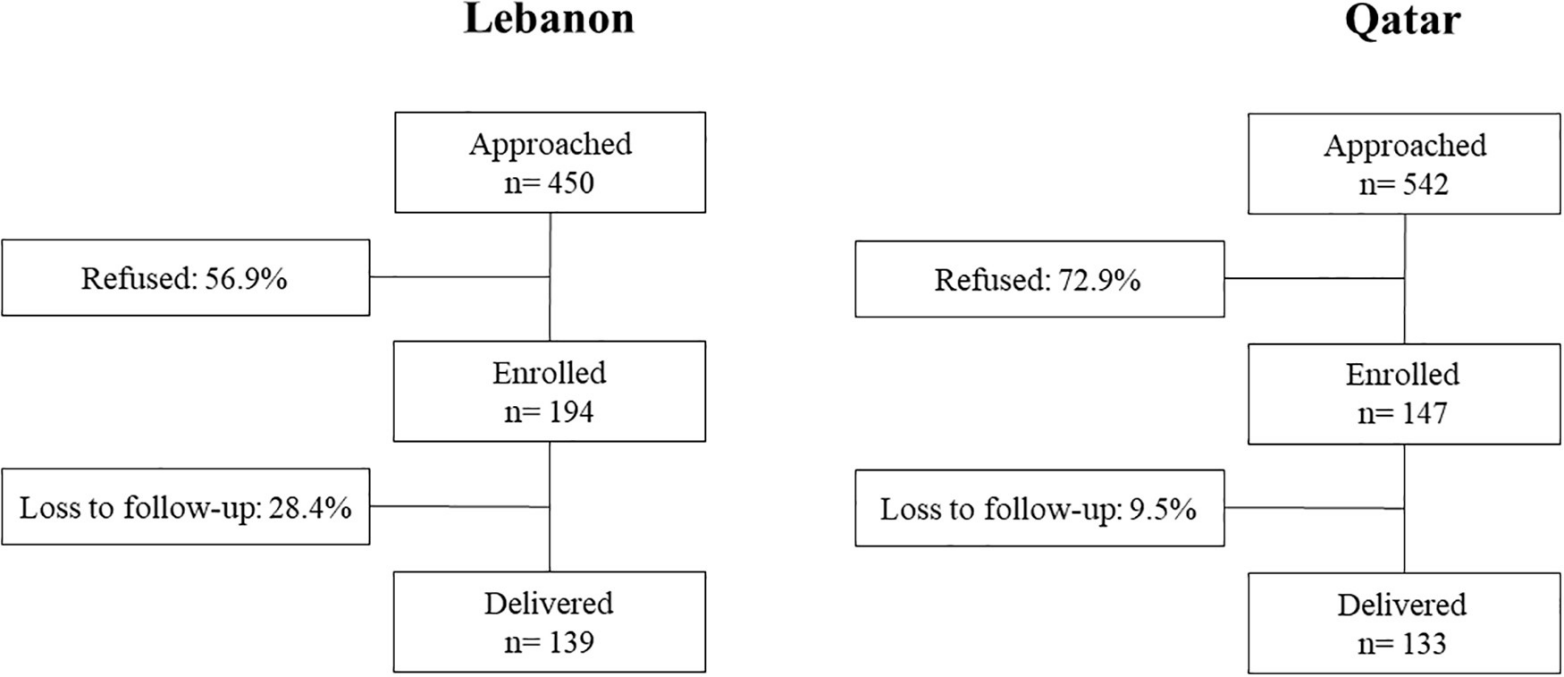
Flow chart of the MINA cohort subjects’ recruitment, in Lebanon and Qatar.

Fig 1 represents the recruitment flow of women in the MINA cohort in Lebanon and Qatar. Overall, 992 eligible pregnant women were approached (n = 450 in Lebanon and n = 542 in Qatar). Of those, 341 (34.4%) accepted to participate, signed the consent form and were enrolled in the cohort (n = 194 in Lebanon and n = 147 in Qatar). Delivery data were available for 272 women (loss to follow up rate: 20.2%; 28.4% in Lebanon and 9.5% in Qatar). The two main reasons which were cited for dropping out were lack of time and moving out of the country. The table describing the associations of various socio-demographic characteristics with loss to follow is found in S1 Appendix. In the overall sample population, loss to follow up was associated with a higher age of the husband (p = 0.03) and a lower income (p = 0.01). In Lebanon, in addition to income, a lower education level of the husband was found to be associated with loss to follow up (p = 0.04). In Qatar, the main determinant of loss to follow up was employment status, whereby employed women were more likely to drop out as compared to women who were housewives (p = 0.04). (S1 Appendix).

Table 1 describes the socio-demographic and lifestyle characteristics of the total study sample, as well as by country. Overall, 40.4% of women were older than 30 years and 22.7% were younger than 25 years of age. For 61.8% of the sample, this pregnancy was not their first. A total of 66.2% had a university degree or higher and 46.3% were employed. In the cohort, 16.3% of women had family ties with their husbands. As for income, 62.7% of women had a high monthly income compared to 14.5% who were in the low income category. With regards to lifestyle characteristics before and during pregnancy, only 5.3% of women reported being current smokers before pregnancy and 2.4% during pregnancy; 41.1% did not consume breakfast regularly before pregnancy and 26.8% during pregnancy. Only 28.1% of participants took folic acid supplementation before pregnancy, while 84.9% did during pregnancy. The comparison of socio-demographic and lifestyle factors between Lebanon and Qatar showed that, compared to pregnant women in the Qatari arm, Lebanese pregnant women were more likely to have a university or higher education level (77.3% vs 51.4%, p<0.001); report a ‘smoker’ status before and during pregnancy (9.3% vs 0%, p<0.001; 4.1% vs 0%, p = 0.01 (respectively)) and take folic acid supplementation before pregnancy (34.6 vs 19.4%, p = 0.002). On the other hand, in the Qatari arm of the cohort, greater proportions of women were not in their first pregnancy (69.4% vs 55.4%, p = 0.01), were housewives (67.1% vs 43.5%, p<0.001), had family ties with their husband (25.5% vs 9.3%, p<0.001, belonged to the high income level (83.9% vs 53.7%, p<0.001) and were not consuming breakfast regularly during pregnancy (36.6% vs 19.5%, p<0.001), as compared to women in the Lebanese arm of the cohort. (Table 1).

**Table 1:** Distribution of socio-demographic and lifestyle characteristics of pregnant women enrolled in the MINA*.

	Total (n = 341)	Lebanese (n = 194)	Qatari (n = 147)	p-value**
**Maternal age (years)**				0.59
18–24.9	77 (22.7)	42 (21.6)	35 (24.1)	
25–29.9	125 (36.9)	76 (39.2)	49 (33.8)	
≥30	137 (40.4)	76 (39.2)	61 (42.1)	
**Number of children**				**0.01**
0	123 (38.2)	78 (44.6)	45 (30.6)	
≥ 1	199 (61.8)	97 (55.4)	102 (69.4)	
**Education**				**<0.001**
Up to high school ⁑	115 (33.8)	44 (22.7)	71(48.6)	
University or higher	225 (66.2)	150 (77.3)	75(51.4)	
**Employment status**				**<0.001**
Employee	156(46.3)	108 (56.5)	48 (32.9)	
Housewife	181(53.7)	83 (43.5)	98 (67.1)	
**Family ties with husband**				**<0.001**
Yes	55(16.3)	18 (9.3)	37 (25.5)	
No	283(83.7)	175 (90.7)	108 (74.5)	
**Total monthly income**				**<0.001**
Low	28(14.5)	24(18.3)	4(6.5)	
Middle	44(22.8)	38(29.0)	6(9.7)	
High	121(62.7)	69(52.7)	52(83.9)	
**Smoking**				
**Pre-pregnancy**				**<0.001**
Non smoker	322(94.7)	176(90.7)	146(100.0)	
Smoker	18(5.3)	18(9.3)	0(0.0)	
**During Pregnancy**				**0.01**
Non smoker	330 (97.6)	186 (95.9)	144 (100)	
Smoker	8 (2.4)	8 (4.1)	0 (0)	
**Breakfast consumption**				
**Pre-pregnancy**				0.56
Not regular	137(41.1)	76(39.8)	61(43.0)	
Regular	196(58.9)	115(60.2)	81(57.0)	
**During Pregnancy**				**<0.001**
Not regular	89(26.8)	37(19.5)	52(36.6)	
Regular	243(73.2)	153(80.5)	90(63.4)	
**Maternal folic acid supplement use**				
**Pre-pregnancy**				**0.002**
No	241(71.9)	125(65.4)	116(80.6)	
Yes	94(28.1)	66(34.6)	28(19.4)	
**During pregnancy**				0.89
No	50(15.1)	28(14.8)	22(15.4)	
Yes	282(84.9)	161(85.2)	121(84.6)	
**Physical activity (MET.hr.week**^**-1**^**)**				0.88
Low	74(32.5)	51(33.1)	23(31.1)	
Moderate	76(33.3)	52(33.8)	24(32.4)	
Vigorous	78(34.2)	51(33.1)	27(36.5)	
**Age of husband (years)**	33.1±6.0	33.5±5.8	32.5±6.3	0.12
**Husband’s education**				0.10
Up to high school ⁑	118 (34.6)	60 (30.9)	58 (39.5)	
University or higher	223 (65.4)	134 (69.1)	89 (60.5)	

Numbers in **bold** face are statistically significant (p-value ≤0.05).

*Values in this table represent mean ±SD and n (%) for continuous and categorical variables, respectively.

**p-values were derived from independent t-test and chi-square test for continuous and categorical variables, respectively, comparing Lebanon and Qatar.

^⁑^ Including technical diploma

Table 2 displays the pregnancy characteristics as well as the birth outcomes in the MINA cohort. Overall, 42.1% of women had a BMI ≥ 25 Kg/m^2^ with higher proportions reported in the Qatari arm as compared to the Lebanese arm (58% vs 30.8%, p<0.001). Only 30.2% of women had adequate GWG, while 25.7% and 44.1% of women had insufficient and excessive GWG, respectively. No significant difference was noted for the distribution of the various categories of GWG between the two arms of the cohort. As for birth outcomes, in the MINA cohort, the mean gestational age was 38.59±1.59 weeks. Mean birthweight and birth length were 3.23±0.49 Kg and 50.15±2.50 cm, respectively. Gestational age and birth length were significantly higher in Qatar compared to Lebanon (p = 0.03 and p = 0.01, respectively). In the cohort, 8.5% of infants were preterm, 5.5% had LBW (<2.5 kg) and 4.3% were macrocosmic (>4.0 Kg). Furthermore, while 68.7% had a weight adequate-for-gestational age (AGA), 6.7% were SGA and 24.6% were LGA. No significant differences were observed for the distribution of birthweight and birthweight for age classification between the Lebanese and Qatari arm of the cohort.

**Table 2:** Pregnancy characteristics and birth outcomes in the MINA cohort *.

	Total	Lebanon	Qatar	p-value**
**Maternal characteristics**				
**Maternal pre-pregnancy BMI**				
<25 Kg/m^2^	183(57.9)	128(69.2)	55(42.0)	**<0.001**
≥25 Kg/m^2^	133(42.1)	57(30.8)	76(58.0)	
**GWG (Kg)**				
Insufficient	52(25.7)	27(21.3)	25(33.3)	0.12
Adequate	61(30.2)	43(33.9)	18(24.0)	
Excessive	89(44.1)	57(44.9)	32(42.7)	
**Birth outcomes**				
**Gestational age (weeks)**	38.59±1.59	38.39±1.31	38.80±1.60	**0.03**
**Birthweight (Kg)**	3.23±0.49	3.24±0.48	3.23±0.51	0.87
**Birth length (cm)**	50.15±2.50	49.77±2.42	50.55±2.53	**0.01**
**Gestational age (weeks)**				
<37 weeks (preterm)	22 (8.5)	10 (7.7)	12 (9.3)	0.64
≥37 weeks	237 (91.5)	120 (92.3)	117 (90.7)	
**Birthweight classification**				0.75
Low birthweight (<2.5 Kg)	14(5.5)	6(4.6)	8(6.4)	
Normal birthweight (2.5–4.0 Kg)	231(90.2)	120(91.6)	111(88.8)	
Macrosomia (>4.0 Kg)	11(4.3)	5(3.8)	6(4.8)	
**Gestational age classification**				0.08
SGA (< 10^th^ centile)	17(6.7)	5(3.9)	12(9.8)	
AGA (10^th^–90^th^ centile)	173(68.7)	87(67.4)	86(69.9)	
LGA (> 90^th^ centile)	62(24.6)	37(28.7)	25(20.3)	

**Abbreviations**: BMI: body mass index; GWG: gestational weight gain; SGA: small-for-gestational age; AGA: adequate-for-gestational age; LGA: large-for-gestational age

Numbers in **bold** face are statistically significant (p-value ≤0.05).

*Values in this table represent mean ±SD and n (%) for continuous and categorical variables, respectively.

**p values were derived from independent t-test and chi-square test for continuous and categorical variables, respectively, comparing Lebanon and Qatar.

**Fig 2:**
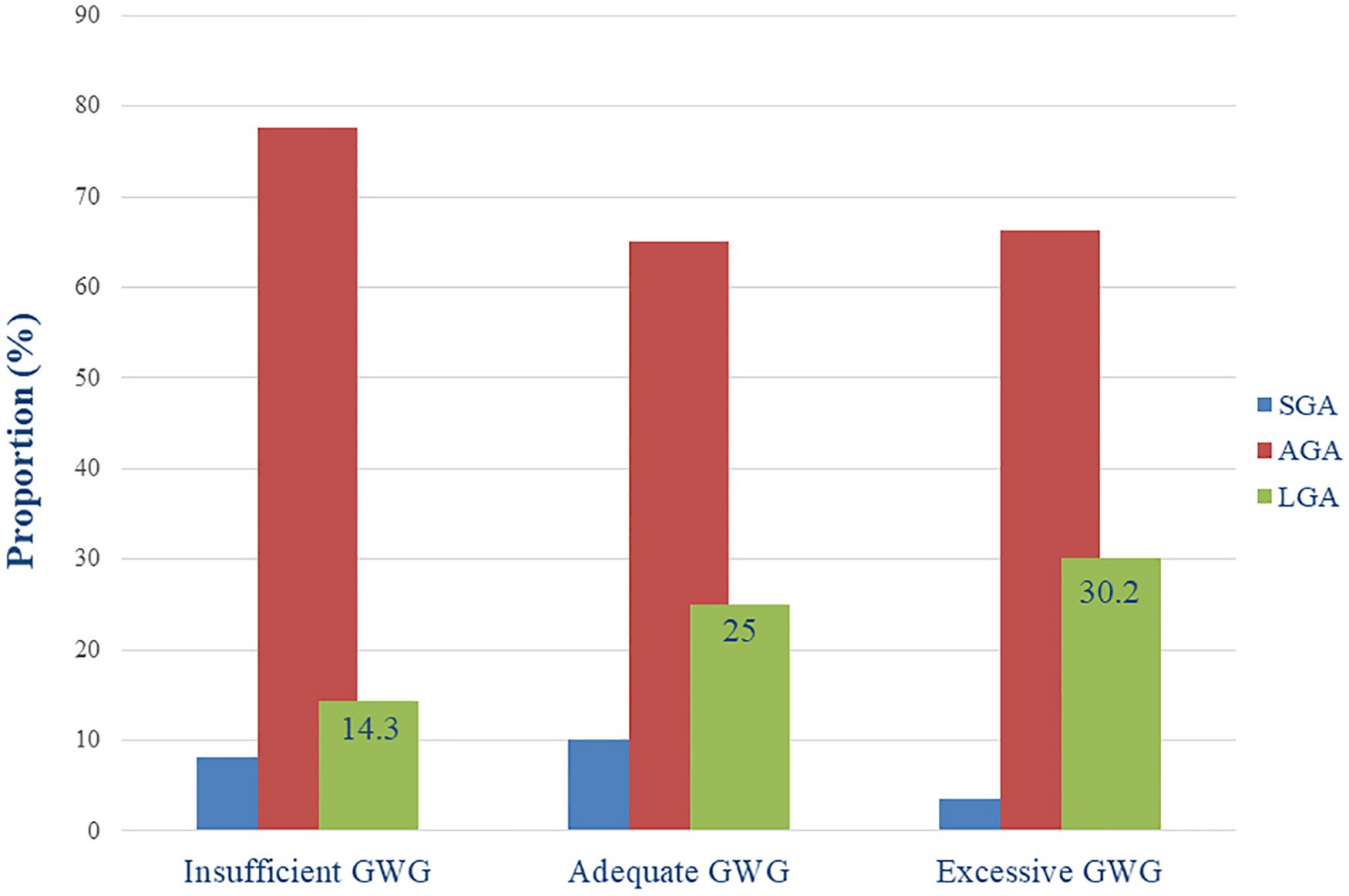
Distribution (%) of SGA, AGA and LGA birthweights according to the various categories of GWG in the study population.

The distribution of the weight for age (SGA, AGA, and LGA) was significantly different across the various categories of GWG in the MINA cohort (p<0.05). (Fig 2). The proportions of LGA were higher with greater GWG. More specifically 14.3%, 25% and 30.2% of infants were classified as LGA among women with insufficient, adequate and excessive GWG, respectively. (Fig 2).

The correlates of pre-pregnancy BMI ≥ 25 Kg/m^2^ were examined among the study participants, using simple and multiple logistic regressions. Simple regression results showed that, among socio-demographic variables, maternal age, nationality, number of children, education levels of mother and husband are all associated with pre-pregnancy BMI. (Table 3). The results of the multiple logistic regression analysis, which included the aforementioned variables that were found significantly associated with the outcomes, showed that women who were 30 years or older were more likely to have a pre-pregnancy BMI ≥ 25 Kg/m^2^ (OR: 3.24, 95% CI: 1.43–7.32). In addition, Qatari women were 3 times more likely to be overweight or obese before pregnancy (OR: 2.85, 95%CI: 1.69–4.81). A higher education level was associated with significantly lower odds of pre-pregnancy BMI ≥ 25 Kg/m^2^ (OR: 0.43, 95%CI: 0.22–0.85).

**Table 3:** Simple logistic regression analysis for the association of study characteristics with a pre-pregnancy BMI ≥25 kg/m^2^ among MINA cohort participants*.

Pre-pregnancy BMI **		
	OR (95% CI)	p-value
**Maternal age (years)**		
18–24.9	Ref.	
25–29.9	1.63(0.87–3.05)	0.13
≥ 30	**2.77(1.50–5.13)**	**0.001**
**Nationality**		
Lebanese	Ref.	
Qatari	**3.10(1.95–4.95)**	**<0.001**
**Number of children**		
0	Ref.	
≥ 1	**2.52(1.54–4.14)**	**<0.001**
**Education**		
Up to high school ⁑	Ref.	
University or higher	**0.44(0.27–0.71)**	**0.001**
**Employment status**		
Employed	Ref.	
Housewife	0.95(0.63–1.49)	0.82
**Family ties with husband**		
No	Ref.	
Yes	1.32(0.71–2.44)	0.38
**Husband’s education**		
Up to high school ⁑	Ref.	
University or higher	**0.60 (0.37–0.96)**	**0.03**
**Total monthly income**		
Low Income	Ref.	
Middle Income	1.10(0.42–2.89)	0.85
High Income	0.68(0.29–1.59)	0.37
**Pre-pregnancy smoking status**		
Non-smoker	Ref.	
Smoker	0.19(0.02–1.57)	0.12
**Pre-pregnancy breakfast consumption**		
Not regularly	Ref.	
Regularly	1.03(0.65–1.63)	0.91

**Abbreviations**: BMI: body mass index

Numbers in **bold** face are statistically significant (p-value ≤0.05).

*Values in this table represent OR and their corresponding 95% confidence intervals

**The reference category is pre-pregnancy BMI <25 kg/m2

^⁑^ Including technical diploma

The results of the associations of GWG with socio-demographic and lifestyle characteristics are presented in Table 4. A higher maternal age (≥ 30 years) was associated with 2.69 times the odds of excessive GWG, 95% CI (1.15–6.31). Women in the Qatari arm were more likely to fall below recommendations for GWG (OR: 2.21, 95%CI: 1.02–4.80). A pre-pregnancy BMI ≥ 25 Kg/m^2^ was associated with 3-fold increase in the odds of excessive GWG, 95% CI (1.56–6.17). In addition, regular breakfast consumption was associated with higher odds of excessive GWG (OR: 2.47, 95%CI: 1.08–5.66). (Table 4). After adjustment for significant determinants, the results of the multiple multinomial regression confirmed that a Qatari nationality was associated with insufficient GWG (p<0.05) (OR: 2.99, 95%CI:1.23–7.25), while pre-pregnancy BMI ≥ 25 Kg/m^2^ and regular breakfast consumption were all significant predictors of excessive GWG (p<0.05). (OR: 3.20, 95%CI: 1.48–6.91; OR: 2.84, 95%CI: 1.15–7.02), respectively).

**Table 4:** Simple multinomial logistic regression analysis for the association of participants’ characteristics with GWG among study participants *.

	Gestational Weight Gain (GWG)**			
	Insufficient GWG	p-value	Excessive GWG	p-value
**Maternal age (years)**				
18–24.9	Ref.		Ref.	
25–29.9	1.64(0.62–4.29)	0.32	2.13(0.91–4.96)	0.08
≥30	2.21(0.85–5.74)	0.10	**2.69(1.15–6.31)**	**0.02**
**Nationality**				
Lebanese	**Ref**		Ref.	
Qatari	**2.21(1.02–4.80)**	**0.04**	1.34(0.67–2.70)	0.41
**Number of children**				
0	Ref.		Ref.	
≥ 1	1.79(0.78–4.07)	0.17	0.77(0.38–1.53)	0.46
**Education**				
Up to high school⁑	Ref.		Ref.	
University or higher	0.62(0.28–1.36)	0.23	.80(0.39–1.63)	0.54
**Employment status**				
Employed	Ref.		Ref.	
Housewife	1.21(0.57–2.56)	0.62	0.61(0.31–1.18)	0.14
**Family ties with husband**				
No	Ref.		Ref.	
Yes	1.89(0.62–5.74)	0.26	1.65(0.60–4.57)	0.34
**Husband’s education**				
Up to high school⁑	Ref.		Ref.	
University or higher	0.57(0.26–1.24)	0.16	1.20(0.58–2.48)	0.62
**Total monthly income**				
Low	Ref.		Ref.	
Middle	0.62(0.12–3.22)	0.57	2.07(0.37–11.53)	0.41
High	0.66(0.15–2.80)	0.57	2.06(0.42–10.11)	0.37
**Smoking During pregnancy**				
Non-smoker	Ref.		Ref.	
Smoker	0.57(0.10–3.26)	0.53	0.32(0.06–1.82)	0.20
**Pre-pregnancy BMI**				
<25 Kg/m^2^	Ref		Ref	
> = 25 Kg/m^2^	0.53(0.22–1.27)	0.15	**3.11(1.56–6.17)**	**0.001**
**Breakfast consumption during pregnancy**				
Not regularly	Ref.		Ref.	
Regularly	1.16(0.50–2.69)	0.74	**2.47(1.08–5.66)**	**0.03**
**Maternal folic acid supplement use**				
No	Ref.		Ref.	
Yes	0.93(0.31–2.76)	0.89	1.13(0.42–3.06)	0.81
**Physical activity (MET.hr.week**^**-1**^**)**				
Low	Ref		Ref	
Moderate	0.83(0.30–2.27)	0.71	0.89(0.37–2.16)	0.80
Vigorous	1.29(0.48–3.44)	0.62	0.96(0.39–2.37)	0.92

**Abbreviations**: BMI: body mass index; GWG: gestational weight gain

Numbers in **bold** face are statistically significant (p-value ≤0.05).

*Values in this table represent OR and their corresponding 95% confidence intervals

**The reference category is “within recommendations”

^⁑^ Including technical diploma

## Discussion

This is the first study to report on results stemming from the MINA cohort [23]. It described the sociodemographic and lifestyle characteristics of the cohort participants in Lebanon and Qatar and examined pre-pregnancy BMI and GWG and their determinants in the study population. It showed that 42% of women in Lebanon and Qatar entered pregnancy with a BMI exceeding 25 kg/m^2^, and only 30% had adequate GWG. High pre-pregnancy BMI was found to be associated with older age, a Qatari nationality and lower education levels. In turn, a higher pre-pregnancy BMI was found to be an independent risk factor for excessive GWG. Noteworthy, the study described the cohort’s birth outcomes, showing that 8.5% of infants were preterm, 5.5% had LBW, 4.3% were macrocosmic, 6.7% were SGA and 24.6% were LGA. A positive association between excessive GWG and LGA was observed.

The study population included 341 pregnant women from Lebanon and Qatar, with some disparities being noted in the subjects’ characteristics between countries. Some of these disparities are a direct reflection of inter-country differences in wealth, cultural aspects, and social norms. For instance, Qatar is one of the richest countries in the world in terms of Gross Domestic Product per capita [29], hence explaining the significantly higher income among Qataris in our study. In addition, the proportions of housewives and those reporting family ties between husband and wife were significantly higher in Qatar, which potentially reflects the prevalent social norms in the country. Consanguinity (i.e. marriage between first cousins) is in fact common in the Qatari context and, approximately a third of women of reproductive age are not employed [29]. The fact that the prevalence of smoking was found to be nill in the Qatari arm of the cohort may be due to a reporting bias, given that smoking is considered as a socially unacceptable behavior among women [30].

In our study, approximately 4 out 10 women (42%) were found to have a high pre-pregnancy BMI, with significantly higher proportions in Qatar (58%) compared to Lebanon (30.8%). The observed proportions among Lebanese women are within the range reported in the literature [31–34], but those observed among Qatari women are considerably higher. Pre-pregnancy overweight, which reflects the mother’s nutritional status prior to conception, may increase the risk of adverse neonatal outcomes [35–38] and heighten the offspring’s risk for chronic diseases later in life [39, 40]. Thus, the observed high proportion of pre-pregnancy overweight in our study may carry long-lasting ramifications on the health and wellbeing of future generations and further exacerbate the NCD epidemic. Therefore, there is an eminent need for culture-specific preventive strategies and a concerted action among various concerned health authorities to foster healthy body weight in women of childbearing age. To be effective, such strategies ought to be based on a thorough understanding of context-specific correlates of pre-pregnancy overweight. In our study, and in line with previous investigations [41–43], higher age (above 30 years) was associated with increased odds of pre-gestation overweight. Older age may in fact be accompanied by multiple pregnancies and consequently a higher possibility of weight retention. Alternatively, it is well known that energy expenditure tends to decrease with age, which may further aggravate the risk of weight gain among women [44, 45]. Another factor that was found to be associated with pre-pregnancy BMI was education level. More specifically, higher educational attainment was associated with significantly lower odds of pre-pregnancy overweight. Education would in fact enable women to obtain information about health, particularly nutrition-related information, and consequently contribute to improving dietary behavior, lifestyle and hence the prevention of overweight [46, 47]. Previous studies conducted in Lebanon and Qatar showed that higher educational attainment was associated with increased adherence to healthier dietary and lifestyle patterns [46, 48], and lower prevalence of obesity and adiposity-related metabolic abnormalities among women [49, 50]. The fact that, in our study, a Qatari nationality was an independent risk factor for pre-pregnancy overweight is reflective of the higher overall prevalence of overweight and obesity in Qatar (and other Gulf Cooperation Council (GCC) countries) [51–53], compared to Lebanon (and the Levant area) [4, 54, 55]. Qatar is in fact categorized as a country in advanced nutrition transition stage, with high levels of overweight and obesity and significant shifts in diet and lifestyle towards westernized patterns, while Lebanon is still categorized as a country in early nutritional transition stages, with relatively moderate levels of obesity [56].

The study findings documented a high prevalence of insufficient and excessive GWG, with no significant differences between Lebanon and Qatar. Overall, only 30% of the participating women fell within the adequate GWG based on the IOM guidelines, while 26% and 44% had insufficient and excessive GWG, respectively. These rates are within the range reported by recent studies, including a systematic review and meta-analysis which showed that 23% and 47% of pregnant women had insufficient and excessive GWG, respectively [31, 57]. The observed high prevalence of inadequate GWG and particularly excessive GWG is of public health concern, given its potential adverse effect on birth outcomes as well as disease risk later in life [58, 59]. Among the predictors of excessive GWG were pre-pregnancy BMI and breakfast consumption. In our study, and in accordance with findings reported by previous studies [11–13], a high pre-pregnancy BMI was found to be an independent risk factor for excessive GWG. These findings have implications on the inter-generational cycle of obesity. Women who have excessive weight gain during pregnancy are more likely to retain their weight post-partum [60] and to enter the next pregnancy with a higher BMI. The latter is by itself a risk factor for another cycle of excessive GWG and the delivery of heavier babies who have higher odds of becoming overweight or obese later in life [61]. These observations may carry long-term public heath ramifications, especially in the local context of the EMR, where the prevalence of overweight and obesity is increasing at an alarming rate [1, 4]. In Qatar, aggressive weight management is being implemented among pregnant women as part of the “Clinical Guidelines for the Prevention, Diagnosis and Management of Diabetes in Pregnancy” that were developed by the Ministry of Public Health [62]. This may explain the fact that in our study, a Qatari nationality was found to be an independent predictor of low GWG. Unexpectedly, regular breakfast consumption was found to be a significant predictor of excessive GWG in our study. Very few studies have examined breakfast consumption in relation of GWG. A review article by Phelan et al (2011) has included regular breakfast consumption within the list of potentially effective interventions for the prevention of excessive GWG [63]. In this context, it is important to note that energy density and nutrient composition may be key confounders for the association between breakfast’s frequency of consumption and excessive GWG. A breakfast high in sugar and refined grains may promote weight gain as opposed to a breakfast that is rich in whole grains, fruits, vegetables and protein [64]. There is therefore a need for future studies that investigate the association of breakfast consumption with GWG while taking into consideration its composition as well as its frequency.

The study has also importantly described the cohort’s birth outcomes. Overall, the rates of preterm birth, LBW, SGA, LGA and macrosomia obtained in this study were comparable to regional and international estimates. More specifically, the rates of preterm deliveries (8.5%) in our study were lower than the 2014 global estimate of 10.6% [65] and the rates reported for Asia as a whole (10.4–13.4%) [65]. Similarly the rates of LBW (5.5%) were below the global estimate of 16% in 2013 [66] and the regional average for the EMR (19.3%) [67]. The observed rate of SGA (6.7%) is in agreement with that reported recently from a birth cohort in Kuwait (7.4%) [68], while the rate of macrosomia (4.3%) was within the range described for both developing (0.5–14.9%) and developed countries (5–20%) [68–70]. As for LGA, its prevalence (24.6%) was similar to that reported from a recent study in Kuwait [68], while being higher than estimates reported from Italy and China (5.3–10.6%) [71, 72]. The high prevalence of LGA may be explained by the prevalence of excessive GWG in our cohort, which is a known risk factor for accelerated fetal growth [31, 73, 74]. In fact, our results documented a significant association between LGA and excessive GWG. The mechanisms behind this association could be related to insulin resistance, which often occurs in women with excessive weight gain during pregnancy [73]. Insulin resistance causes metabolic abnormalities that increase the flux of nutrients to the fetus, including large amounts of glucose, resulting in hyperinsulinemia and fetal growth acceleration [73]. Insulin resistance may also be associated with higher concentrations of triglycerides that placental lipases hydrolyze and transfer to fetal circulation, resulting in increased energy input to the fetus [73, 75, 76].

A secondary objective of this study was the investigation of the socio-demographic determinants of loss to follow-up in the MINA cohort. Our results showed that loss to follow up was associated with certain socioeconomic factors such as lower income, but also with specific characteristics among husbands, including older age and lower education status. These findings resonate with those obtained by a recent qualitative study conducted by our group among pregnant women in Lebanon, and which has investigated the interaction between individual and contextual factors in motivating or deterring pregnant women from participating in research studies [77]. In that study, partners were recognized as levers whose full support would incite women to be more engaged in the research experience, whereas their lack of support can hinder the subjects’ interest and deter their participation [77]. In line with our study findings, many of the participants in the qualitative study pointed to education level as an important factor in the decision to participate in research studies, while misconception and lack familiarity with research was described as an important deterrent [77]. The fact that women’s employment status was associated with loss to follow up in our study is in agreement with previous studies highlighting concerns around time commitment, and work absenteeism as major barriers against participation in research studies [77, 78].

The strengths of this study include its prospective nature which allows for the exploration of causal relationships whilst requiring less recall than other epidemiological study designs [79, 80]. In addition, though the MINA cohort is multi country, the protocols and procedures were standardized throughout data collection and cleaning. Modelled after the protocols of the MINA was another cohort in the region: the Mother and Infant Study Cohort (MISC) in the Northern Emirates of the UAE [81]. The unified methodology in these cohorts will allow for cross country comparisons in the future. However, the results of this study ought to be considered in light of the following limitations. First the low response rate observed in our study may have potentially led to a selection bias, while the small sample size may have led to underpowered analyses. Second, information pertinent to pre-pregnancy BMI and GWG were extracted from the participants’ medical records. Although standards techniques were implemented by the clinics and health care centers for the measurement of body weight, we cannot rule out the possibility of random errors in these measurements. Third, the information pertinent to socio-demographic and lifestyle characteristics were collected using a questionnaire in an interview setting. As observed in the majority of questionnaire-based studies, the interview-based approach may be associated with social desirability bias [82]. However, in our study, fieldworkers had received extensive training prior to data collection in order to reduce judgmental verbal and nonverbal communication and hence minimize potential social desirability bias.

## Conclusion

The high prevalence of pre-pregnancy overweight and excessive GWG among the MINA participants raises major public health concern, given the mounting evidence for their association with maternal and neonatal complications as well as adverse health outcomes later in life. The study showed that pre-pregnancy BMI was an independent predictor of excess GWG and that the latter was associated with higher rates of LGA. Importantly, this study identified the sociodemographic determinants of high pre-pregnancy BMI and excessive GWG in the local context of countries in the EMR. These findings should feed into the development of culture-specific evidence based interventions for the promotion of healthy body weight in women of childbearing age, and the prevention of excessive weight gain during pregnancy. The adoption of heathy diets, and appropriate sleep and physical activity patterns ought to be integrated within health care programs that target women in countries of the EMR.

## Supporting information

S1 Appendix Socio-demographic determinants of loss to follow up in the MINA cohort. (DOCX)

S2 Appendix Data used in the study. (XLSX)
