# Supplementary material for: Pre-pregnancy BMI, gestational weight gain and birth outcomes in Lebanon and Qatar: Results of the MINA cohort
Source: PLoS One. 2019 Jul 2;14(7):e0219248. doi: 10.1371/journal.pone.0219248 (PMC6605672; doi:10.1371/journal.pone.0219248)
Supplement: S1 Appendix — (DOCX) [file pone.0219248.s001.docx]

**Supporting information**

**S1 Appendix**. **Socio-demographic determinants of loss to follow up in the MINA cohort^*^**

|  | **Total** | | |  | **Lebanon** | | |  | **Qatar** | |  |
| --- | --- | --- | --- | --- | --- | --- | --- | --- | --- | --- | --- |
|  | **MINA participants who delivered (n=272)** | **Participants lost to follow up (n=69)** | **p-value^**^** |  | **MINA participants who delivered (n=139)** | **Participants lost to follow up (n=55)** | **p-value^**^** |  | **MINA participants who delivered (n=133)** | **Participants lost to follow up (n=14)** | **p-value^**^** |
| **Maternal age (years)** | 28.5±5.3 | 28.8±5.6 | 0.61 |  | 28.6±5.5 | 29.0±5.8 | 0.59 |  | 28.4±5.1 | 28.0±5.1 | 0.80 |
| **Age of husband (years)** | 32.7±6.0 | 34.4±5.8 | **0.03** |  | 33.0±5.7 | 34.7±5.7 | 0.06 |  | 32.4±6.4 | 33.1±6.2 | 0.72 |
| **Number of children** |  |  |  |  |  |  |  |  |  |  |  |
| 0 | 93 (36.0) | 30 (46.9) | 0.11 |  | 52 (41.6) | 26 (52.0) | 0.21 |  | 41 (30.8) | 4 (28.6) | 0.86 |
| ≥ 1 | 165 (64.0) | 34 (53.1) |  |  | 73 (58.4) | 24 (48.0) |  |  | 92 (69.2) | 10 (71.4) |  |
| **Education** |  |  |  |  |  |  |  |  |  |  |  |
| Up to high school ⁑ | 96 (35.4) | 19 (27.5) | 0.22 |  | 29 (20.9) | 15 (27.3) | 0.34 |  | 67 (50.8) | 4 (28.6) | 0.11 |
| University or higher | 175 (64.6) | 50 (72.5) |  |  | 110 (79.1) | 40 (72.7) |  |  | 65 (49.2) | 10 (71.4) |  |
| **Employment status** |  |  |  |  |  |  |  |  |  |  |  |
| Employee | 121 (45.1) | 35 (50.7) | 0.41 |  | 81 (59.6) | 27 (49.1) | 0.19 |  | 40 (30.3) | 8 (57.1) | **0.04** |
| Housewife | 147 (54.9) | 34 (49.3) |  |  | 55 (40.4) | 28 (50.9) |  |  | 92 (69.7) | 6 (42.9) |  |
| **Family ties with husband** |  |  |  |  |  |  |  |  |  |  |  |
| Yes | 48 (17.8) | 7 (10.1) | 0.12 |  | 13 (9.4) | 5 (9.1) | 0.94 |  | 35 (26.7) | 2 (14.3) | 0.31 |
| No | 221 (82.2) | 62 (89.9) |  |  | 125 (90.6) | 50 (90.9) |  |  | 96 (73.3) | 12 (85.7) |  |
| **Husband’s education** |  |  |  |  |  |  |  |  |  |  |  |
| Up to high school ⁑ | 89 (32.7) | 29 (42.0) | 0.15 |  | 37 (26.6) | 23 (41.8) | **0.04** |  | 52 (39.1) | 6 (42.9) | 0.78 |
| University or higher | 183 (67.3) | 40 (58.0) |  |  | 102 (73.4) | 32 (58.2) |  |  | 81 (60.9) | 8 (57.1) |  |
| **Total monthly income** |  |  |  |  |  |  |  |  |  |  |  |
| Low | 16 (10.6) | 12 (28.6) | **0.01** |  | 12 (18.3) | 12 (34.3) | **0.02** |  | 4 (7.3) | 0 (0) | 0.71 |
| Middle | 34 (22.5) | 10 (23.8) |  |  | 29 (30.2) | 9 (25.7) |  |  | 5 (9.1) | 1 (14.3) |  |
| High | 101 (66.9) | 20 (47.6) |  |  | 55 (57.3) | 14 (40.0) |  |  | 46 (83.6) | 6 (85.7) |  |

Numbers in **bold** face are statistically significant (p-value ≤0.05).

^*^Values in this table represent mean±SD and n (%) for continuous and categorical variables, respectively.

^**^p-values were derived from independent t-test and chi-square test for continuous and categorical variables, respectively.

⁑ Including technical diploma
